# Differentiation-specific action of orphan nuclear receptor NR5A1 (SF-1): transcriptional regulation in luteinizing bovine theca cells

**DOI:** 10.1186/1477-7827-4-64

**Published:** 2006-12-19

**Authors:** Norbert Walther, Martina Jansen, Wasima Akbary, Richard Ivell

**Affiliations:** 1 Institute for Hormone and Fertility Research, University of Hamburg, Falkenried 88, D-20251 Hamburg, Germany; 2 School of Life Science Hamburg, University Hospital Eppendorf, Hamburg, Germany; 3 Research Unit Molecular Oncology, Clinic for General Surgery and Thoracic Surgery, Christian-Albrechts-University, Kiel, Germany; 4 Allergopharma Joachim Ganzer KG, Reinbek, Germany; 5 School of Molecular and Biomedical Science, University of Adelaide, Adelaide, Australia

## Abstract

**Background:**

The orphan nuclear receptor NR5A1 (steroidogenic factor-1, SF-1) is a master regulator of tissue-specific gene expression in reproductive and steroidogenic tissues. Two activating functions, AF-1 and AF-2, have been described to function in a cooperative manner to recruit transcriptional coactivators to the promoter regions of NR5A1-controlled genes.

**Methods:**

The role of the NR5A1 activating functions AF-1 and AF-2 was studied in primary bovine theca cells. Bovine theca cells were infected with recombinant adenovirus vectors over-expressing wild-type NR5A1 or NR5A1 mutants, in which one of the activating functions of this orphan nuclear receptor had been impaired. Under different culture conditions, theca cell-specific transcript levels were measured by reverse transcription and real-time PCR.

**Results:**

Under culture conditions optimized for cell growth, transcriptional up-regulation of CYP11A1 (P450 side chain-cleavage enzyme) and INSL3 (Insulin-like factor 3, Relaxin-like factor (RLF)) was found to be dependent on the presence of NR5A1 carrying an intact AF-2. Under conditions inducing luteal differentiation of theca cells, CYP11A1 and STAR (Steroidogenic acute regulatory protein) were up-regulated by the action of luteinizing hormone (LH), whereas the differentiation-specific up-regulation of INSL3 was suppressed by LH in luteinizing theca cells. Inhibition of insulin- or IGF1- (insulin-like growth factor I) dependent signal transduction by the RAF1 kinase inhibitor GW5074 and the mitogen-activated protein kinase kinase inhibitor PD98059 resulted in the finding that RAF1 kinase inhibition was able to counteract the LH-dependent regulation of NR5A1-controlled genes, whereas inhibition of the mitogen-activated protein kinase (MAP kinase) pathway did not have any significant effect.

**Conclusion:**

The regulation of the three NR5A1-controlled genes CYPA11, STAR, and INSL3 in luteinizing theca cells apparently is not dependent on NR5A1 activating functions AF-1 or AF-2. Activation of AF-1 here even appears to have an impairing effect on NR5A1 transcriptional activity, implying that up-regulation of NR5A1-controlled genes uses a different pathway. Our results might be explained by the possible existence of an interconnection between the RAF1 kinase and the cyclic AMP-protein kinase A pathway. Such a non-classical regulatory pathway might play an important role in the control of gene expression in reproductive and steroidogenic tissues.

## Background

The various differentiated phenotypes of cells and tissues are based upon the establishment of stable cell type-specific patterns of gene expression. The recent advances in the sequencing of mammalian genomes [1], together with the development of efficient high-throughput technologies for quantification of gene products [2,3], allows the assessment of the tissue-specific expression levels for virtually all genes in a single experiment. Although data on gene expression can now be generated with high efficiency, sophisticated molecular biology techniques like chromatin immunoprecipitation [4] have brought to light completely new levels of complexity in the mechanisms controlling transcription [5,6]. The elucidation of the regulatory functions of nuclear receptors represents a good example of the step-by-step detection of further levels of complexity. The nuclear receptor proteins purified first could be described as transcriptional activators controlled by small lipophilic molecules as ligands [7-9]. However, related proteins could be characterized, whose transcriptional activity apparently is not primarily controlled by ligand binding [10,11]. These orphan nuclear receptors are evolutionary old molecules with essential roles in the regulation of development and tissue function. The principal effect of binding of these proteins to the promoters of controlled genes appears to be the generation of specific docking sites for transcriptional coregulators. The transcriptional activity of orphan nuclear receptors can be regulated by phosphorylation [12,13]. Although recently in a number of cases small lipophilic molecules have been shown to be able to bind as ligands to orphan nuclear receptors [14-17], the functions of these compounds in vivo have not been well established. An additional level of transcriptional regulation by orphan nuclear receptors is the alternative binding to the same promoter sites [18,19]. Binding of different orphan nuclear receptors can lead to the recruitment of different coactivators or corepressors to the promoter of the controlled genes [20,21]. The subsequent process of gene activation comprises the establishment of an open chromatin conformation and the assembly of the RNA polymerase II transcriptional initiation complex [22-24]. The activities of the coactivators in this process, like the transcriptional activities of the orphan nuclear receptors, can be under the control of protein phosphorylation, modulating the specific interactions of coactivators with different transcriptional initiation complexes [25].

For the orphan nuclear receptor NR5A1, the carboxyterminal activating function AF-2, corresponding to the ligand-dependent activating function of nuclear hormone receptors, has been shown to be responsible for binding of co-activators of the p160 class providing an essential function for the constitutive transcriptional activity of NR5A1 [20]. The unique activating function AF-1 of NR5A1 located in the hinge region of the protein is regulated by mitogen-activated kinase (MAPK) phosphorylation and is involved in interactions with various cofactors and in stabilization of the AF-2 activating function [12,26,27]. In the light of the complexity of the mechanisms regulating transcription, the multifaceted effects of hormones and growth factors on the development and function of tissues are not surprising. For example, ovarian follicular cells are under cyclic control by the differentiation-inducing gonadotropins follicle stimulating hormone (FSH) and luteinizing hormone (LH), in combination with essential growth-promoting effects by hormones and growth factors like insulin and/or IGF1 (insulin-like growth factor I) [28,29]. Tissue culture of bovine theca cells provides an excellent model system to investigate the influences of these endocrine and paracrine stimuli on the development of the differentiated phenotype or the induction of theca cell differentiation-specific genes [30]. Synergistic activation by LH and IGF1 appears to provide the full stimulus for luteal differentiation of ovarian follicular cells [31,32]. Many of the genes induced or up-regulated during the luteinization process, for example the genes coding for the steroidogenic P450 enzymes, the Steroid Acute Regulatory Protein (*STAR*) and the Insulin-like factor 3 (*INSL3*) appear to be controlled by binding of the tissue-specific orphan nuclear receptor NR5A1 to specific binding sites in the promoter regions [33-39]. In the study presented here, the regulatory mechanisms controlling expression of these luteinization-specific genes were investigated, using in vitro cultures of bovine theca cells under conditions optimized for growth or differentiation.

Our results suggest that the established activating functions of NR5A1 only play a minor role in the control of reproductive functions during theca cell luteinization. Possibly, in luteinizing bovine theca cells an interconnection exists between the MAP kinase pathway and the cyclic AMP-protein kinase A pathway, constituting a non-classical pathway of growth factor and hormone action.

## Methods

### Plasmids, bacteria, cell lines, and chemicals

Expression vectors for full-length wild-type *NR5A1 *cDNAs (pRSV/Ad4BP (bovine *NR5A1*) and pCMV119+ (murine *NR5A1*)) were kind gifts of Dr. K.I. Morohashi (Okazaki, Japan) and Dr. K. Parker (Dallas, TX), respectively. The bovine AF-2 deletion mutant (pAd4BP/ΔAF-2) was kindly provided by Dr. J. Lund (Bergen, Norway), the murine AF-1 mutant S203A (pCI-HA-NR5A1) was generously supplied by Dr. H.A. Ingraham (San Francisco, CA). The components of the AdEasy adenoviral expression system, plasmids pAdEasy-1 and pAdTrack-CMV, as well as the rec^+ ^bacterial strain E. coli BJ5183, were obtained from Dr. T. Wieland (Mannheim, Germany) with the kind permission of Dr. B. Vogelstein (Baltimore, MD). HEK 293 cells for adenovirus propagation were a gift of Dr. F. Schnieders (Hamburg, Germany), HeLa cells were obtained from the American Type Culture Collection. Both cell lines were cultivated in Dulbecco minimum essential medium supplemented with 2 mM L-glutamine, 100 IU/ml penicillin, 100 μg/ml streptomycin, and fetal calf serum (10 % for HeLa cells, varying amounts ranging from 2 % to 5 % for HEK 293 cells). Protein kinase inhibitors H89 and PD98059 were purchased from Calbiochem (San Diego, CA), inhibitor GW5074 and all other chemicals were from Sigma (Munich, Germany).

### Construction of NR5A1-expressing recombinant adenoviruses

All recombinant DNA experiments were performed using standard laboratory protocols [40]. Wild-type bovine *NR5A1 *cDNA was directly recloned into shuttle plasmid pAdTrackCMV [41] as a HindIII/XbaI fragment. The cDNA coding for the AF-2 deletion mutant was released by digestion and blunting at the BamHI site, followed by SalI cleavage; the resulting fragment was inserted into SalI/EcoRV-digested pAdTrackCMV. Kanamycin-resistant plasmid clones were analysed by NdeI cleavage. Wild-type murine *NR5A1 *cDNA was isolated as an EcoRI fragment and, after subcloning into pBSSK (Stratagene, Heidelberg, Germany), released by digestion with KpnI and XbaI and inserted into appropriately cleaved pAdTrackCMV. The cDNA coding for the S203A mutant was directly recloned as XhoI/NotI fragment into SalI/NotI-digested pAdTrackCMV. Identity and orientation of *NR5A1 *sequences was confirmed by dideoxy sequencing. As an empty virus vector control pAdTrackCMV was used without any further modification.

For generation of complete adenoviruses by homologous recombination, the *NR5A1 *wild-type and mutant shuttle plasmids were linearized by PmeI digestion and purified by agarose gel electrophoresis to minimize the contamination with circular plasmid DNA. 80 to 1200 ng of these DNAs, together with 100 ng of undigested plasmid pAdEasy-1 comprising the necessary additional adenoviral sequences [41], were transfected into rec^+ ^E. coli BJ5183 by electroporation. Cuvettes with 2 mm distance between electrodes were used with the Gene Pulser System (BIORAD, Munich, Germany) at settings of 2500 V, 25 μF, and 200 Ω, resulting in time constants of the pulse of approximately 5 msec. Directly after electroporation, bacteria were incubated at 37°C for 1 hour with shaking and spread on kanamycin-containing plates. Recombinant adenoviral plasmid clones were selected after 16 hours at 37°C as small colonies. Plasmid DNA from these clones was analysed by digestion with PacI, plasmids containing the expected 3 kb PacI fragment were retransformed into rec^- ^E. coli for further analysis and preparation of adenoviral DNA. The identity of recombinant adenovirus constructs was confirmed by diagnostic PCR using different combinations of primers derived from adenovirus vector or *NR5A1 *sequences and by dideoxy sequencing.

### Preparation of infectious adenovirus particles

Recombinant adenoviral plasmid DNAs were linearized by PacI digestion leading to the excision of plasmid sequences and used for adenovirus propagation. Four to 25 μg of adenovirus DNAs were transfected into HEK 293 cells grown in T25 tissue culture flasks to approximately 70 % confluence by lipofection using the Polyfect reagent (Qiagen, Hilden, Germany) according to the manufacturer's instructions. Virus expression was monitored by fluorescence microscopy of the coexpressed green fluorescent protein. Seven to 10 days after transfection, cells were scraped off the flask and cells and supernatants were subjected to three cycles of freezing and thawing for release of virus particles from infected cells. Cellular debris was removed by centrifugation (5 min, 3800 rpm) and total supernatants were used for infection of T75 flasks of HEK 293 cells grown to 70 % confluence. After 2 days virus suspensions were prepared from the infected cells and total supernatants used for infection of T175 flasks of HEK 293 cells as described above. The titer of the virus suspensions prepared from these cells was determined by limiting dilution using HEK 293 cells. For the final step of adenovirus propagation, 5 T300 tissue culture flasks were seeded each with 2 × 10^7 ^HEK 293 cells and the cells infected with recombinant adenoviruses at a multiplicity of infection of 25 (5 × 10^8 ^plaque-forming units per flask). Approximately 2 days after infection, when infected cells just began to detach from the plate, supernatants were carefully removed. The cells were scraped off the flask and subjected to freeze/thaw lysis as described above. After centrifugation, 5 ml each of the virus suspensions were layered on top of CsCl step gradients (2 ml 1.45 g/cm^3^, 2 ml 1.35 g/cm^3^, 1 ml 1.25 g/cm^3^) in Beckman SW40 ultracentrifuge tubes. Tubes were filled up and tared with mineral oil and centrifuged for 2 hours at 35,000 rpm and 10°C. After removal of cellular and viral debris, intact virus particles were collected using a 1 ml syringe and transferred to new SW40 tubes. Tubes were filled up with CsCl solution (1.35 g/cm^3^), tared with mineral oil, and centrifuged for 20 hours at 30,000 rpm and 4°C. The virus band was collected as described above and desalted by passage through a NAP-25 gel filtration column (Amersham Pharmacia Biotech, Frankfurt, Germany) using virus storage buffer (10 mM Tris/HCl, pH 8.0, 135 mM NaCl, 3 mM KCl, 1 mM MgCl_2_, 10 % glycerol). The virus suspensions were divided into aliquots and stored at -80°C. The titer of the frozen stocks was determined by limiting dilution using HEK 293 cells.

### Preparation and culture of bovine theca cells

For preparation of bovine theca cells, the established protocol described previously by Brunswig-Spickenheier and Mukhopadhyay [30] was used. Briefly, bovine ovaries were collected at a local slaughterhouse and healthy tertiary follicles with a diameter of 10 to 25 mm were used for dissection of the theca interna layer. After enzymatic dissociation of the tissue, theca cells were purified by centrifugation over Percoll (density 1.07 g/cm^3^). Theca cells were suspended in a 1:1 mixture of Dulbecco minimum essential medium and Ham F12 medium containing 2 mM L-glutamine, 100 IU/ml penicillin, 100 μg/ml streptomycin (basal medium) and 5 × 10^5 ^cells per well seeded into 6-well tissue culture plates (Nunc, Wiesbaden, Germany) coated with bovine dermal collagen (Cellon, Strassen, Luxembourg). For the investigation of growing cultures, the basal medium was supplemented with 2.5 % fetal calf serum. After 2 days of culture at 37°C under an atmosphere of 95 % air and 5 % CO_2_, the medium was changed to basal medium supplemented with 10 μg/ml insulin, 5 μg/ml transferrin, 5 ng/ml sodium selenite, and 0.1 % bovine serum albumin. For long-term cultures of differentiating theca cells, the basal medium was supplemented with 100 ng/ml insulin, 5 μg/ml transferrin, 5 ng/ml sodium selenite, and 0.1 % bovine serum with or without the addition of 10 ng/ml bovine luteinizing hormone (bLH, kind gift of the NIADDK and the National Hormone and Pituitary Program, NIH, Bethesda, MD); the medium was changed in regular intervals every 2 days.

### Infection of bovine theca cells with NR5A1 expressing recombinant adenoviruses

Prior to adenovirus infection, medium was changed on theca cell cultures adding only 1 ml of basal medium per well. Diluted virus stocks were then added with a multiplicity of infection of 100 (5 × 10^7 ^plaque-forming units per well) and evenly distributed by gentle shaking immediately after addition. After incubation for 30 min, 1.5 ml of basal medium and the necessary supplements were added and the infected cells cultured for another 2 or 3 days. Adenovirus infections of growing theca cells were performed at day 5 of culture, infections of long-term cultures were performed at days 5, 8, and 11. Virus expression was monitored by fluorescence microscopy of the coexpressed green fluorescent protein. For isolation of total cellular RNA, cells were lysed 48 to 72 hours after infection in 500 μl peqGOLD RNAPure reagent (Peqlab, Erlangen, Germany) per well and RNA purified following the manufacturer's instructions and stored at -80°C. For isolation of total cellular protein, cells were lysed in 250 μl RIPA buffer (10 mM Tris-HCl pH 7.2, 150 mM NaCl, 0.1 % SDS, 1.0 % Triton X-100, 1.0 % Na-deoxycholate, 1 × Complete Protease Inhibitor Cocktail (Roche Diagnostics, Penzberg, Germany)). After removal of cellular debris by centrifugation, protein extracts were aliquoted and stored at -80°C.

### Quantification of theca cell-specific transcripts

1 to 2 μg total bovine theca cell RNA were reverse transcribed using SuperScript II RNase H^- ^reverse transcriptase (Invitrogen, Karlsruhe, Germany) and random hexamer oligonucleotide primers (Amersham Biosciences, Freiburg, Germany) according to the manufacturer's instructions. cDNAs were diluted 1 : 5 with ultra-pure water and stored at -20°C. For amplification of theca cell-specific transcripts, real time-PCR measurements were performed in triplicate on the LightCycler System (Roche Diagnostics). PCR reactions were set up using the QuantiTect SYBR Green PCR Kit (Qiagen) and specific oligonucleotide 5' and 3' primers (10 pmol each). Primer sequences for amplification of partial cDNA sequences of theca cell-specific genes were selected using the Primer3 PCR primer picking program [42]. The primer sequences used are listed in Tab. 1. For initial denaturation and activation of the DNA polymerase, samples were heated to 95°C for 15 min. Amplifications were performed over up to 40 cycles (95°C 15 sec (denaturing), 50°C 30 sec (annealing), 72°C 15 sec (polymerisation), 75°C 5 sec (data acquisition)). The specificity of the reactions was monitored by melting curves of the products and agarose gel electrophoresis, and the identity of the products confirmed by dideoxy sequencing. Standard curves for measurements of PCR efficiencies were generated using serial 1 : 5 dilutions of pooled theca cell cDNAs. Expression of the ribosomal protein RPS27A was used as an internal control for relative transcript quantification. For the calculation the method of Pfaffl [43] was used, including an automatic correction for differences in PCR efficiencies. Testing for statistical significance of the differences in transcript levels was performed by one-way analysis of variance (ANOVA) followed by the Newman-Keuls test using the GraphPad Prism 4.0 software package (GraphPad Software Inc., San Diego, CA); P < 0.05 was considered statistically significant.

**Table 1:** Primer sequences used for amplification of bovine theca cell transcripts

**Transcript**	**5' Primer**	**3' Primer**	**Product size**
bovine *RPS27A*	CTGGCAAGCAACTGGAAGAT	TCTTTTCTTAGCGCCACCAC	104 bp
bovine *CYP11A1*	ACTTTCGCCACATCGAGAAC	CACGTCTTCAGGGTGAATGAT	98 bp
bovine *STAR*	CTCAAGGACCAAACTCAC	ATTGGCAAAATCCACCTG	106 bp
bovine *INSL3*	CGCTGGTCTTCCGAGGAG	CCCATGGAGGAGATGTTGTC	90 bp

### Fluorescence microscopy

GFP fluorescence of adenovirus-infected theca cells was visualised by fluorescence microscopy using a Diaphot microscope (Nikon, Düsseldorf, Germany) equipped with a DM510 filter block and a 10DIC lens at 100-fold magnification. Digital images were recorded using a Leica DC200 camera (Leica Microsystems, Wetzlar, Germany) and stored as JPEG files for documentation.

### Northern blot and Western blot analyses

Total cellular RNA or protein was isolated 48 hours after infection. In vitro translation of NR5A1 proteins was performed using the TnT rabbit reticulolysate system (Promega, Mannheim, Germany). Northern blot analysis of adenovirus-infected HeLa cells was performed as described previously [44]. DNA fragments comprising bovine *NR5A1 *or human ribosomal protein *RPS27A *coding sequences were radiolabelled and used as probes. For Western blot analysis 6 μg of total cellular proteins were separated on a 10 % NuPAGE gel (Invitrogen, Karlsruhe, Germany) at 200 V and blotted to a PVDF membrane at 30 V following the manufacturer's instructions. After blocking with 5 % non-fat milk powder, as primary antibody a rabbit antiserum directed against the bovine NR5A1 protein (kind gift of K.I. Morohashi, dilution 1 : 5000) was allowed to bind over-night at 4°C. After washing, the second antibody (horseradish-peroxidase-conjugated anti-rabbit IgG (Dianova, Hamburg, Germany) was added at a dilution of 1 : 5000 and allowed to bind for 3 hours at room temperature. After final washing, binding of antibodies was analyzed using the Amersham ECL Plus detection system (GE Healthcare, Munich, Germany).

## Results

### Adenoviral vectors for over-expression of NR5A1 wild-type and mutants

The orphan nuclear receptor NR5A1 is one of the most important regulators of tissue-specific gene expression in reproductive and steroidogenic tissues. Although the binding of this tissue-specific transcription factor to regulatory sites in the promoters of numerous genes expressed in these tissues has been established [39], the mechanisms by which NR5A1 exerts control of transcription remain largely unelucidated. In the study presented here, an experimental system has been established that allows the analysis of NR5A1 action during growth and differentiation of gonadal cells. Bovine theca cells were cultivated under different in vitro conditions and infected with recombinant adenovirus vectors over-expressing wild-type NR5A1 or NR5A1 mutants, in which one of the activating functions of this orphan nuclear receptor had been impaired. This approach allowed us to measure separately the effects mediated by the individual NR5A1 activating functions on the transcription of tissue-specifically expressed genes in theca cells during growth and differentiation.

**Figure 1:**
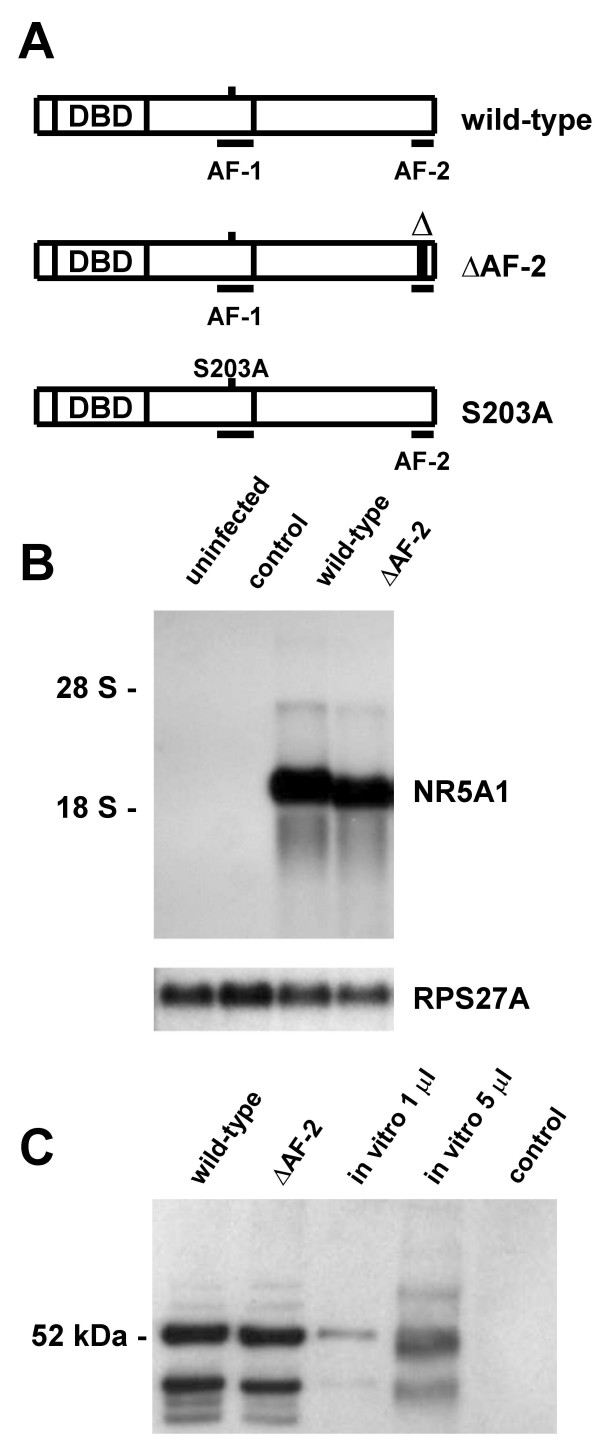
**Transactivation domains of NR5A1 wildtype and mutants over-expressed in bovine theca cells**. In the ΔAF-2 mutant the essential core region of the AF-2 activating function has been deleted. In the AF-1 mutant amino acid serine 203 has been substituted by alanine preventing the MAP kinase-dependent phosphorylation of this site (**A**). Northern blot analysis of wild-type and mutant mRNAs transcribed from NR5A1-expressing recombinant adenoviruses in infected HeLa cells (Control: AdEasy adenoviral vector without insert). As a control for equal RNA loading a probe specific for human ribosomal protein RPS27A was used (**B**). Western blot analysis of NR5A1 protein from adenovirus-infected HeLa cells and rabbit reticulocyte lysate in vitro translation using an antiserum directed against bovine NR5A1 (Control: AdEasy adenoviral vector without insert) (**C**).

NR5A1 has been shown to contain two activating functions. A mutant carrying a deletion of the core region of the AF-2 activating function has been shown to suppress completely the activation of steroidogenic enzyme expression [45]. The recently defined unique AF-1 activating function of NR5A1 has been shown to be regulated by phosphorylation of amino acid serine 203 [12] leading to a stabilization of the active conformation of NR5A1 [27]. Complete cDNAs for wild-type bovine as well as murine *NR5A1*, a mutant carrying the described deletion of the AF-2 core region (ΔAF-2), and a mutant carrying a substitution of the phosphorylatable amino acid serine 203 by alanine (S203A) (Fig. 1A) were used for generation of recombinant adenoviruses over-expressing these different NR5A1 proteins in infected cells.

In the AdEasy adenovirus vector system [41] used in this study the inserted genes of interest are expressed to high levels of mRNA from the strong ubiquitously active cytomegalovirus immediate early promoter. Infections of HeLa cervical carcinoma cells were performed to obtain mRNA amounts large enough to perform Northern blot analysis to check for size and stability of the transcripts (Fig. 1B). *NR5A1 *cDNA sequences are expressed to high levels of stable mRNAs whose sizes correspond well with the sizes of the open reading frames contained within the cloned inserts. Adenovirus vectors expressing *NR5A1 *wild-type and mutant mRNAs were further tested for NR5A1 protein expression. In parallel, Western blot analyses of infected HeLa cervical carcinoma cells using an antiserum specific for bovine NR5A1 [46] were performed, demonstrating the expression of a major protein band with an estimated molecular weight of approximately 52 kDa comigrating with bovine NR5A1 protein in vitro translated from the original plasmid expression vector (Fig. 1C).

### Over-expression of NR5A1 wild-type and mutants in growing bovine theca cells

**Figure 2:**
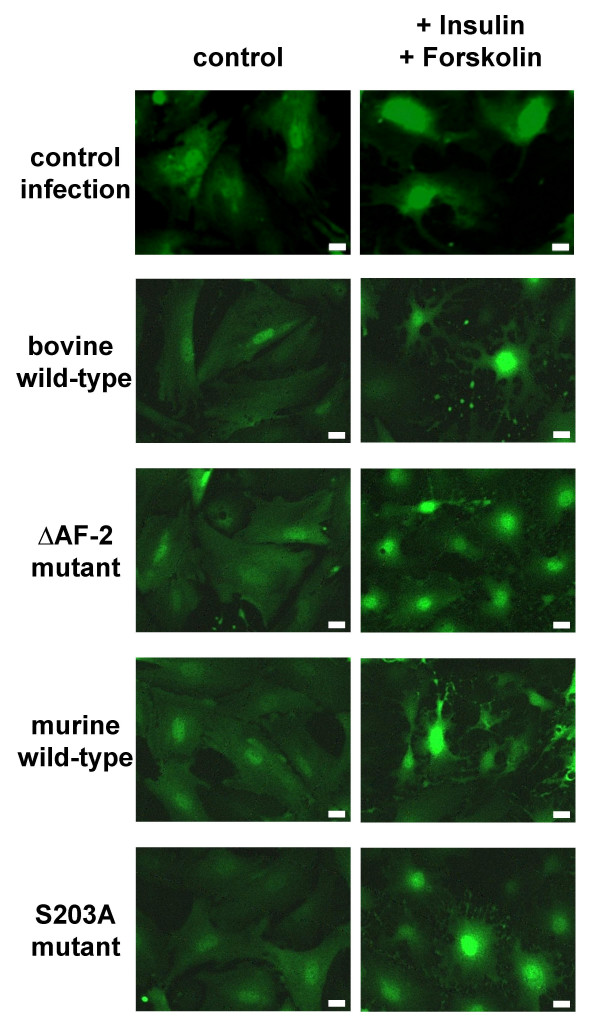
**GFP fluorescence of bovine theca cells infected with adenoviral vectors expressing NR5A1**. Bovine theca cells were infected with adenoviral vectors expressing wild-type or mutant NR5A1 and stimulated or not (control) with 2 μg/ml insulin and 10 μM forskolin (Insulin/Forskolin). The scale bar represents 20 μm.

The main stimulus inducing luteal differentiation of theca cells is the secretion of luteinizing hormone (LH) to high levels around the time of ovulation. The acute cellular response of theca cells to this stimulus is the increase in the intracellular concentration of the second messenger cyclic AMP, leading within hours to a total reorganization of the actin cytoskeleton of the cell generally referred to as "stellation", which is characterized by cytoplasmic retraction and the formation of narrow, elongated cellular processes [47]. This response was investigated in bovine theca cells infected with recombinant adenoviruses over-expressing wild-type or mutant NR5A1 (Fig. 2). Infected cells were stimulated or not with a combination of insulin and the adenylate cyclase activator forskolin and visualised by the fluorescence of the green fluorescent protein coexpressed from the recombinant adenoviruses. Although the morphology of the theca cells undergoing "stellation" is variable, nearly all cells in the cultures show this response, the cytoplasmic retraction being indicated by the increased level of perinuclear fluorescence. The results show that previous infection and over-expression of wild-type or mutant NR5A1 did not influence the acute cellular response of bovine theca cells to the luteinizing stimulus.

**Figure 3:**
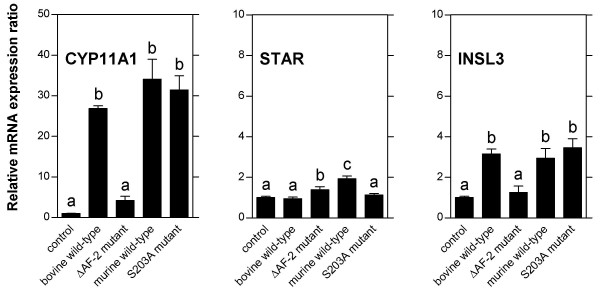
**Transcriptional regulation by NR5A1 of theca cell marker genes in growing cultures**. Bovine theca cells cultivated in the presence of 10 μg/ml insulin for maximal growth were infected with adenoviral vectors expressing bovine or murine wild-type NR5A1 (WT) or mutants lacking one of the two activating functions (ΔAF-2 or S203A, control: AdEasy adenoviral vector without insert). *CYP11A1*, *STAR*, and *INSL3 *transcripts were quantified by real time-PCR as described in Materials and Methods. The results of one representative out of three experiments are shown. Data are expressed as mean ± standard deviation. The groups designated by the letters above the bars differ significantly at the level of P < 0.05.

The results of the initial experiments demonstrate that infection with recombinant adenoviruses at a multiplicity of infection of 100 induces expression of wild-type and mutant NR5A1 to high levels in bovine theca cells without toxic effects and without disturbing intracellular responses. The high multiplicity is needed in order to obtain infection of 100 % of the cells in the culture, probably due to variations in the number of virus receptors on the surfaces of individual theca cells. Adenovirus infections of bovine theca cells were performed to elucidate the contribition of the two NR5A1 activating functions to the transcriptional regulation of theca cell-specifically expressed genes. Growing cultures of bovine theca cells were infected at day 5 of culture, total RNA isolated 2 days after infection, and relative transcript levels quantificated by real time-PCR (Fig. 3). Both the bovine and the murine *NR5A1 *wild-type forms were used because of the different genetic backgrounds of the two *NR5A1 *mutants investigated. The results show for the steroidogenic enzyme gene *CYP11A1*, a classical NR5A1-regulated gene [35,48], a massive up-regulation of transcription by over-expression of wild-type NR5A1, indicating that this gene is not fully activated by the endogenous NR5A1. This transcriptional activation is completely abolished in the cultures over-expressing the NR5A1 mutant lacking the AF-2 activating function (ΔAF-2), known to be important for coactivator binding. Western blot analysis of NR5A1 proteins confirmed that the mutant protein was expressed to levels identical to those of wild-type NR5A1 (see Fig. 1C). On the contrary, mutation of the MAP kinase phosphorylation site (S203A), known to be essential for AF-1 activating function, has no effect whatsoever on the transcriptional activation of CYP11A1 induced by NR5A1 over-expression. This finding suggests that in bovine theca cells, in contrast to other experimental systems [12], MAP kinase phosphorylation of the NR5A1 AF-1 activating function does not effect NR5A1 function. The observed pattern of AF-2 dependent activation is not restricted to the steroidogenic enzyme gene *CYP11A1*. The transcriptional activation of the tissue-specific growth factor *INSL3 *(Insulin-like factor 3) by over-expressed NR5A1 follows the same pattern, albeit at lower levels. This NR5A1-controlled promoter [39] apparently is regulated by the same principal mechanism that is governing *CYP11A1 *expression. Transcription from the promoter of the *STAR *(Steroidogenic acute regulatory protein) gene does not follow this pattern of activation by NR5A1; this gene does not appear to be affected by NR5A1 over-expression. The differences between the *STAR *expression in the cultures over-expressing bovine or murine wild-type NR5A1, although statistically significant, have been found to be not reproducible in parallel experiments. The promoter of the bovine *STAR *gene can be up-regulated by NR5A1 over-expression in transfection experiments [37]. Several sequences in the bovine *STAR *promoter show reasonably good matches with the established NR5A1 binding site, but binding of NR5A1 to the bovine *STAR *promoter was found to be relatively weak in this study. In the light of these findings, the results presented here indicate that transcription of the *STAR *gene, despite its expression in the same tissues as *CYP11A1*, may not be regulated by direct binding of NR5A1 to the promoter and subsequent coactivator recruitment, but by a different, yet uncharacterized, mechanism.

### Over-expression of NR5A1 wild-type and mutants in long-term cultures of differentiating bovine theca cells

**Figure 4:**
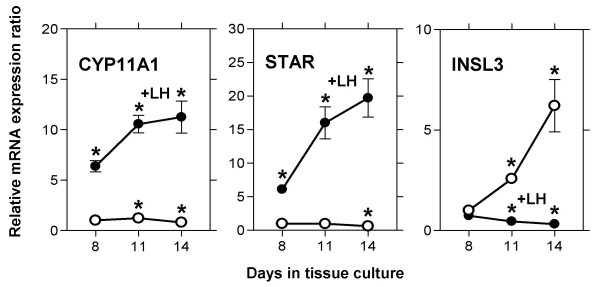
**Time-course of marker gene expression during differentiation of bovine theca cells in culture**. Long-term cultures of bovine theca cells containing minimal amounts of insulin (100 ng/ml) were set up with (filled circles) or without (open circles) the addition of 10 ng/ml bovine luteinizing hormone (LH). At the time-points indicated, *CYP11A1*, *STAR*, and *INSL3 *transcripts were quantified by real time-PCR as described in Materials and Methods. The results of one representative out of three experiments are shown. Data are expressed as mean ± standard deviation. Significant differences (P < 0.05) to the unstimulated cultures at day 8 are indicated by asterisks.

In order to investigate the transcriptional regulation of theca cell-specifically expressed genes under conditions that resemble better their up-regulation in vivo, long-term cultures of bovine theca cells were set up in the presence or the absence of LH as the natural luteinizing stimulus (Fig. 4). Under these established conditions, the time-course of luteal differentiation of theca cells can be observed in tissue culture [44]. The results show that, in spite of the different regulatory effects of NR5A1 on the *CYP11A1 *and the *STAR *promoter, expression of both genes is totally dependent on stimulation by LH and is increasing during prolonged culture, following essentially identical time courses. On the contrary, expression of *INSL3 *slowly decreases during prolonged culture under the influence of LH. In cultures without addition of LH, however, *INSL3 *expression starts to rise after more than 8 days, with levels still increasing after 14 days of culture, confirming the results of a previous study [44]. Apparently in this case, in spite of the same mechanism of NR5A1 regulation of the *INSL3 *and *CYP11A1 *promoters in growing cells, the expression of *INSL3 *during theca cell luteinization is controlled by a principally different mechanism.

**Figure 5:**
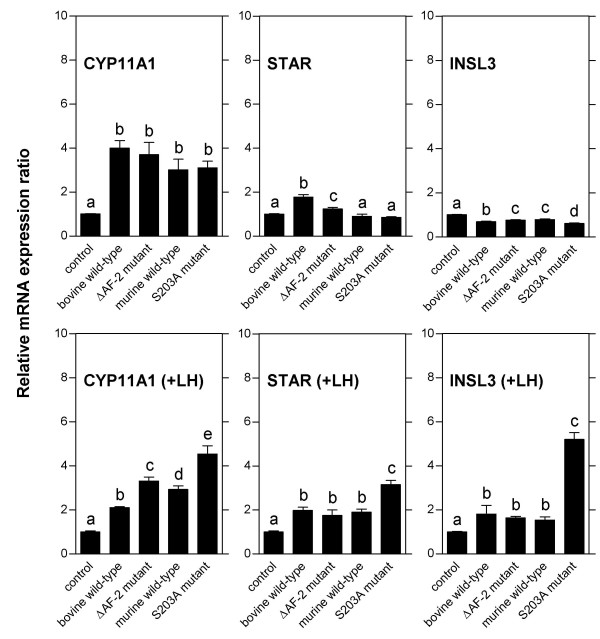
**Transcriptional regulation by NR5A1 of theca cell marker genes during differentiation in culture**. Bovine theca cells were cultivated for 11 days in the presence of minimal amounts of insulin (100 ng/ml) with or without the addition of 10 ng/ml bovine luteinizing hormone (LH) and subsequently infected with adenoviral vectors expressing bovine or murine wild-type NR5A1 or mutants lacking one of the two activating functions (ΔAF-2 or S203A, control: AdEasy adenoviral vector without insert). 72 hours after infection, *CYP11A1*, *STAR*, and *INSL3 *transcripts were quantified by real time-PCR as described in Materials and Methods. The transcript levels in the NR5A1-infected cells are presented in relation to the levels in control-infected cells under identical culture conditions (with or without addition of LH). Thus the bars represent the transcriptional effects of over-expression of NR5A1 wild-type or mutants independent of the stimulatory or inhibitory effects of LH. The results of one representative out of three experiments are shown. Data are expressed as mean ± standard deviation. The groups designated by the letters above the bars differ significantly at the level of P < 0.05.

Infections of long-term cultures of differentiating bovine theca cells (days 5, 8, and 11 of culture) were performed in order to analyze the effects of NR5A1 wild-type and mutant over-expression. At day 11, expression of *CYPA11 *and *STAR *was induced by LH 8-fold and 16-fold, respectively, whereas the induction of *INSL3 *was totally abolished by addition of the hormone, leading to a 6-fold reduction of the *INSL3 *expression level (see Fig. 4). Results are presented for the infections at day 11 (Fig. 5), the infections at day 5 and 8 yielded essentially the same results. The presentation of the normalized results of the infection experiments levels out the stimulation or inhibition of expression by LH, displaying solely the transcriptional effects of NR5A1 over-expression. In the cultures without addition of LH, robust elevation of transcript levels by NR5A1 over-expression could be observed for *CYP11A1 *demonstrating the function of the adenovirally expressed NR5A1. Interestingly, this increase was found in all cultures, irrespective of the status of the activating functions of the over-expressed NR5A1 proteins. Apparently the established activating functions AF-1 and AF-2 of NR5A1 both are not essential for the exertion of its constitutive transcriptional activation under these conditions. The variations between the cultures in the levels of *STAR *and *INSL3 *expression, although statistically significant, have been found to be not reproducible in parallel experiments, suggesting no specific effect of NR5A1 on expression of these genes in the absence of LH. The relative expression levels of theca cell-specific transcripts in the cultures with addition of LH and infected with adenoviruses expressing bovine or murine wild-type NR5A1 or the mutant lacking the AF-2 activating function were variable and, in the case of *CYP11A1 *transcripts, not reproducible in parallel experiments. The elevation of *STAR *and *INSL3 *expression levels in these cultures was maximally two-fold compared to LH-stimulated control cultures. However, infection of theca cells with the AF-1 activating function mutant unable to be phosphorylated by MAP kinases consistently led to a higher induction of *CYP11A1*, *STAR*, and *INSL3 *transcription. This suggests that phosphorylation at this site might have a detrimental effect on the potency of NR5A1 as a transcriptional activator in certain promoter or cellular contexts. This effect is most clearly demonstrated at the *INSL3 *promoter, which in the presence of LH is down-regulated. Presumably, in the differentiating theca cells, NR5A1 phosphorylation at serine 203 leads to the inactivation of a yet uncharacterized activating function of this protein. Our finding differs substantially from the effects of the NR5A1 activating function AF-1 described in other experimental systems. This effect of serine 203 phosphorylation will have to be investigated in further studies.

### Investigation of the role of protein kinase pathways in the regulation of NR5A1-controlled genes

**Figure 6:**
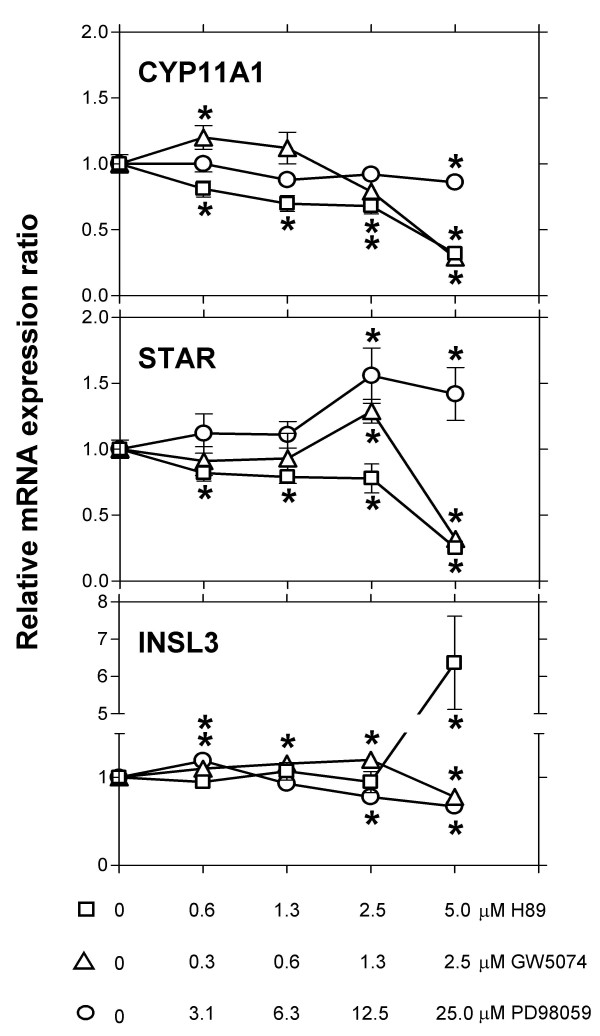
**Involvement of protein kinase pathways in the regulation of marker genes in differentiating theca cells**. Bovine theca cells were cultivated for 11 days in the presence of minimal amounts of insulin (100 ng/ml) and 10 ng/ml bovine luteinizing hormone and subsequently treated with different amounts of specific inhibitors for protein kinase A (H89, squares), RAF1 kinase (GW5074, triangles) or MAP kinase-kinase (PD 98059, circles). After 72 hours, *CYP11A1*, *STAR*, and *INSL3 *transcripts were quantified by real time-PCR as described in Materials and Methods. The results of one representative out of three experiments are shown. Data are expressed as mean ± standard deviation. Significant differences (P < 0.05) to the uninhibited control cultures are indicated by asterisks.

For investigation of the role of the MAP kinase pathway in regulation of NR5A1 function, experiments were performed aimed at the dissection of the kinase cascade constituting this regulatory pathway in bovine theca cells. Long-term cultures of bovine theca cells after 11 days of culture in the presence of LH were treated with specific inhibitors for RAF1 kinase (GW5074) or MAP kinase-kinase (PD98059) as sequential steps of the MAP kinase pathway, or an inhibitor for the cyclic AMP-dependent protein kinase A (H89) (Fig. 6). No cytotoxic effects of the inhibitors were observed over the concentration range employed in these experiments. Addition of the protein kinase A inhibitor H89, as expected, led to a down-regulation of *CYP11A1 *and *STAR *expression, the inhibitor counter-acting the intracellular effects of the exposure of the cells to exogenous LH. The dramatic increase in *INSL3 *expression under these conditions suggests that *INSL3 *expression is actively suppressed by a protein kinase A-dependent mechanism under conditions of LH stimulation.

A consistently observed result of this experiment is the inhibitory effect exerted by the RAF1 kinase inhibitor GW5074 on the expression of both the *CYP11A1 *and the *STAR *gene, in sharp contrast to the lack of any major effects of the MAP kinase-kinase inhibitor PD98059. The lack of correlation between the inhibition of sequential steps of the MAP kinase signal transduction pathway suggests that this pathway may not exist as an isolated entity in theca cells. The effect exerted by RAF1 kinase inhibition strongly correlates with the inhibition effected by the protein kinase A inhibitor H89. Possibly, in differentiating bovine theca cells there exists an interconnection between the MAP kinase and the cyclic AMP-protein kinase A pathways. Such an interconnection would allow RAF1 kinase to stimulate protein kinase A by phosphorylation of some upstream signalling component. This pathway of signal transduction would not be totally unprecedented, as RAF1 kinase-mediated phosphorylation of several adenylyl cyclase isoforms has recently been described in HEK 293 cells [49]. Our results show that such an interconnection between the two signalling pathways may be of importance in regulating luteal differentiation of theca cells. Further studies using complementary methods will help to elucidate this non-classical regulatory mechanism.

## Discussion

The orphan nuclear receptor NR5A1 has been shown to be one of the master regulators controlling the development and function of reproductive and steroidogenic tissues [50]. NR5A1-regulated genes as a whole are controlled by numerous different regimes of hormonal or developmental control [51], producing the high diversity of cellular phenotypes in the reproductive system. For example, the chromatin structure of the promoter region and the presence or absence of tissue-specifically expressed coregulators, both of fundamental importance for the regulation of gene expression in differentiation processes [52,53], are not taken into account in standard transient transfection studies. On the contrary, bovine theca cells in primary culture as a population of steroidogenic cells from a reproductive tissue have been shown to express differentiated features during long-term culture under serum-free conditions [30]. Therefore these cells have been used in the study presented here for the investigation of NR5A1 function in a model system resembling theca cell luteinization in vivo. The use of adenoviral vectors for NR5A1 over-expression in bovine theca cells enabled us to transfer NR5A1 mutants with only one of the two activating functions impaired, and to study the effect of AF-1 and AF-2 on the expression of tissue-specifically expressed genes that are regulated during theca cell luteinization.

Three NR5A1-controlled genes whose expression had been shown to be regulated specifically during luteinization of bovine theca cells, *CYP11A1 *[54], *STAR *[37], and *INSL3 *[44], were selected for quantification of transcript levels. The up-regulation of *CYP11A1 *and *STAR *could be shown to be dependent on the presence of LH during long-term culture, in concordance with previous findings. The lack of major effects of NR5A1 on *STAR *expression, in contrast to the results of experiments using acute LH stimulation of bovine theca cells [56], suggests that, at this promoter, only the direct transcriptional effects of LH may be mediated by NR5A1, whereas the differentiation-specific up-regulation investigated here uses a different regulatory mechanism. NR5A1-mediated, LH-dependent up-regulation of transcription has been shown previously for the gene coding for the neuropeptide hormone oxytocin in the other type of steroidogenic cell in the bovine ovary, the granulosa cell [57]. The oxytocin promoter could be shown by us to be occupied by NR5A1 under this condition of active gene expression [18,58,59], demonstrating the important role of NR5A1 in the LH-dependent up-regulation of transcription. In contrast to the LH-dependency of *CYP11A1 *and *STAR *expression, *INSL3 *up-regulation during long-term culture of bovine theca cells was found to be impaired under LH stimulation. This finding is in perfect correlation with the results of our preceding study [44], confirming that *INSL3 *expression during bovine theca cell differentiation appears to be regulated by a different mechanism than the one controlling the expression of this gene in the male gonad. In the testis, *INSL3 *expression is up-regulated cell type-specifically in the steroidogenic Leydig cells, but in contrast to the situation in luteinizing bovine theca cells, this up-regulation is stimulated by LH [60].

The LH-dependent up-regulation of *INSL3 *expression in mouse Leydig cells appears to be dependent on binding of NR4A1 (NGFI-B) to a specific binding site in the *INSL3 *promoter [61]. This orphan nuclear receptor is involved in the acute LH-dependent up-regulation of gene expression in granulosa cells around the time of ovulation, and its expression apparently persists in the cells of the corpus luteum [62]. However, the NR4A1 binding site in the *INSL3 *promoter appears not to be conserved in all species, being absent in the bovine (Walther and Ivell, unpublished results). Apparently, there exist species-specific differences in the details of the mechanisms regulating *INSL3 *gene expression. The investigation of the contributions of the two NR5A1 activating functions, AF-1 and AF-2, to the control of tissue-specific gene expression in theca cells yielded some unexpected results: Firstly, a comparison of the transcriptional effects of AF-2 between growing bovine theca cells and bovine theca cells luteinizing in long-term cultures demonstrated that, although AF-2 is necessary for NR5A1-dependent up-regulation of gene expression in growing cells, this activating function apparently is dispensible for the exertion of the stimulatory effects of NR5A1 on gene expression during bovine theca cell luteinization. The second result was the total lack of effect of serine 203 phosphorylation on NR5A1 transcriptional activity. This finding implies that the MAP kinase pathway does not contribute directly to the activation of NR5A1-controlled genes by targeting the AF-1 activating function. However, luteal differentiation of bovine theca as well as granulosa cells has been shown to be dependent on stimulation of tyrosine receptor kinases by insulin or IGF1 [28,44,63]. These stimuli in many types of cells result in activation of the MAP kinase signal transduction pathway and subsequent activation of transcription factors like activator protein-1(AP-1) [64]. A possible explanation of our findings is that this pathway may not be functional in luteinizing bovine theca cells and that the undoubted stimulatory effects of insulin or IGF1 on these cells are exerted by a different pathway. Corresponding results have been obtained in a study investigating STAR gene expression in mouse Leydig cells, where the majority of the stimulatory effects of IGF1 are exerted via the protein kinase C pathway [65].

## Conclusion

The results of our experiments suggest that, although the orphan nuclear receptor NR5A1 is necessary for the expression of tissue-specifically expressed genes that control the reproductive functions of the ovary, the established activating functions of NR5A1 play only a minor role, if any, in the control of gene expression during theca cell luteinization. Apparently, in luteinizing theca cells a different mechanism is involved in the regulation of NR5A1-controlled genes. The results of our experiments using specific inhibitors for the MAP kinase and the cyclic AMP-protein kinase A pathway might be explained by the possible existence of an interconnection between these two signal transduction pathways. The recent finding that RAF1 kinase can mediate the phosphorylation of several adenylyl cyclase isoforms [49] demonstrates that such interconnections exist and may be functional depending on cell type or differentiation state. The regulatory role of NR5A1 under these conditions appears to be somewhat reduced to the provision of coactivator docking sites. Transcriptional regulation of NR5A1-controlled genes in luteinizing theca cells presumably is exerted by specific modifications in the composition of coactivator complexes or modifications of the coactivators themselves. The main aim of this study using adenoviral expression of NR5A1 wild-type and mutants was to elucidate the contribution of the NR5A1 activating functions AF-1 and AF-2 to the transcriptional regulation of luteinization-specific genes. For subsequent studies, knock-down of the expression of the involved genes by short interfering RNA (siRNA) will be helpful in analyzing the details of the differentiation-specific mechanisms governing the expression of NR5A1-controlled genes.

In addition to the regulatory mechanisms discussed above, future studies on the regulation of orphan nuclear receptor-controlled genes will have to take into account the highly dynamic formation and destruction of promoter-nuclear receptor-coactivator complexes [5,6]. This new level of complexity demands the development of new experimental methods in order to advance the classical models of transcriptional regulation. Evidence is accumulating that the intracellular signal transduction pathways do not exist in the cell as isolated entities, but are interconnected to an intricate regulatory network. These interconnections previously referred to as "crosstalk" apparently are of outmost importance as essential constituents of this network. RAF1 kinase presumably assumes a central role in interconnecting the "growth promoting" MAP kinase pathway to the "differentiation promoting" cyclic AMP-protein kinase A pathway [66], thereby contributing to the unimpaired development and function of reproductive tissues.

## Competing interests

The author(s) declare that they have no competing interests.

## Authors' contributions

NW conceived the study, constructed the adenoviral expression vectors, established and performed the quantification of transcripts by real time-PCR, and drafted the manuscript. MJ prepared and tested the adenovirus stocks and established the theca cell cultures for infection experiments. WA performed long-term cultures and adenovirus infection experiments. RI participated in the design of the study and the long-term cultures of differentiating theca cells.
